# Evaluation of a walking school bus program: a cluster randomized controlled trial

**DOI:** 10.1186/s12966-024-01602-w

**Published:** 2024-05-10

**Authors:** Ashleigh M. Johnson, Chuan Zhou, Miriam Haviland, Jason A. Mendoza

**Affiliations:** 1 School of Exercise and Nutritional Sciences, San Diego State University, 5500 Campanile Drive, San Diego, CA 92182 USA; 2 Center for Child Health, Behavior, and Development, Seattle Children’s Research Institute, 1920 Terry Avenue, Seattle, WA 98101 USA; 3 Department of Pediatrics, University of Washington, 4800 Sand Point Way NE, Seattle, WA 98105 USA; 4 IQVIA, 101 Elliott Ave W, Seattle, WA 98119 USA; 5 Public Health Sciences, Fred Hutchinson Cancer Center, 1100 Fairview Ave N, Seattle, WA 98109 USA

**Keywords:** Physical activity, Randomized controlled trail, Walking school bus, Youth, School

## Abstract

**Background:**

The purpose of this study was to investigate the effects of a walking school bus intervention on children’s active commuting to school.

**Methods:**

We conducted a randomized controlled trial (RCT) in Houston, Texas (Year 1) and Seattle, Washington (Years 2–4) from 2012 to 2016. The study had a two-arm, cluster randomized design comparing the intervention (walking school bus and education materials) to the control (education materials) over one school year October/November – May/June). Twenty-two schools that served lower income families participated. Outcomes included percentage of days students’ active commuting to school (primary, measured via survey) and moderate-to-vigorous physical activity (MVPA, measured via accelerometry). Follow-up took place in May or June. We used linear mixed-effects models to estimate the association between the intervention and outcomes of interest.

**Results:**

Total sample was 418 students [M_age_=9.2 (SD = 0.9) years; 46% female], 197 (47%) in the intervention group. The intervention group showed a significant increase compared with the control group over time in percentage of days active commuting (β = 9.04; 95% CI: 1.10, 16.98; *p* = 0.015) and MVPA minutes/day (β = 4.31; 95% CI: 0.70, 7.91; *p* = 0.02).

**Conclusions:**

These findings support implementation of walking school bus programs that are inclusive of school-age children from lower income families to support active commuting to school and improve physical activity.

**Trail registration:**

This RCT is registered at clinicaltrials.gov (NCT01626807).

**Supplementary Information:**

The online version contains supplementary material available at 10.1186/s12966-024-01602-w.

## Introduction

Promoting youth physical activity (PA) is a public health priority, as youth PA levels in the United States (US) continue to decline [1, 2]. It is estimated that over 75% of US youth fail to meet PA recommendations [3], and children from low socioeconomic status households typically have lower PA levels [4]. One approach for promoting youth PA is through active commuting to school (ACS), making it easier for youth to fit PA into their daily lives and establish long term routines [5]. ACS (e.g., cycling, walking) can significantly contribute to children’s PA levels and is associated with higher levels of moderate-to-vigorous intensity PA (MVPA) [6]. There lies a strong potential for ACS to improve youth PA levels, and increasing the proportion of students that walk or bike to school is a national health goal [7]. ACS has also been associated with lower measures of adiposity among school-age children [8]. Although a vast majority of students need to commute to school, the prevalence of ACS has declined from about 48% in 1970 to 11% in 2017 [9–11]. The National Household Travel Survey showed that among US school-age children, those in the highest income category (over $100,000 USD) had 1.56 greater odds of walking or biking to school (*p* = 0.002) compared to the lowest income category ($0-$30,000 USD) [10]. Additionally, children with parents with a high school education had significantly lower odds of walking or biking to school compared to those with parents with a college degree [10]. However, the prevalence of ACS among US children is still relatively low [10, 12]. 

The decline in ACS has been partially attributed to perceived lack of safety [13–16]. However, walking with an adult can reduce child pedestrian injury risk by almost 70% [16–19]. The walking school bus (WSB), which involves adults accompanying children during ACS, is a promising intervention that addresses safety concerns while promoting youth PA. WSB also provides teaching opportunities around pedestrian safety skills to and from school, and has been shown to improve child self-efficacy, parent self-efficacy, and parent outcome expectations related to ACS [20]. Both self-efficacy (i.e., an individual’s belief in their ability to complete a task) and outcome expectations (i.e., the anticipated consequences of engaging in a behavior) are associated with youth physical activity [21]. Further, previous research has shown that child and parental self-efficacy and parental outcome expectations are associated with children’s ACS [22–24]. 

A systematic review and meta-analysis of ACS interventions identified WSB as a simple and effective type of ACS intervention [25]. Four WSB interventions found increased ACS following intervention [23, 26–28]. In a 2-week pilot randomized controlled trial (RCT) involving 12 students ages 8–11 years in California, USA, participants were randomized to either being driven to school or in the WSB group. In this study, the authors found no significant group differences for total daily or weekday PA or percentage of time spent in MVPA, which was likely due to insufficient power to detect such differences [26]. A two-year quasi-experimental trial of a WSB intervention was conducted among 324 students aged 6–10 years in Nebraska, USA [28]. Findings showed a significant increase in daily PA between the intervention and control groups (78.0 versus 60.6 min/day), although the evaluation used a non-randomized study design [28]. We conducted the first cluster RCT of a WSB in Texas, USA among 149 students ages 9–10 and showed improvements to rates of children’s ACS and MVPA over 4–5 weeks. Results from this pilot RCT showed that intervention participants significantly increased their weekly percentage of ACS by 37.8% versus controls over time and accelerometer-determined MVPA by 7.0 min/day versus controls over time, although there was a small sample size and a brief intervention period [23]. There remains a gap in the literature as to the long-term efficacy of WSB programs and their impact on rates of ACS, PA, and adiposity. We sought to help fill this gap and build upon our previous short-term pilot study by conducting a cluster RCT of a WSB program over a longer time frame and among a larger number of schools. No known cluster RCTs have reported on long-term efficacy of WSB programs on children’s ACS, MVPA, and weight status. Further, few studies have included children from diverse socioeconomic or racial/ethnic backgrounds. Children living in low-income neighborhoods or who are of racial/ethnic minority backgrounds often experience more active transportation barriers such as lack of access to safe walking routes [29, 30]. Evaluating WSB programs in these communities can help identify effective strategies to overcome these barriers and reduce health disparities.

Our objective was to conduct a cluster RCT of a WSB to examine its impact on children’s ACS and objectively measured PA. We had the following hypotheses: H1) the WSB program would increase children’s ACS over a schoolyear, H1a) parents’ outcome expectations and self-efficacy would mediate the relationship between the WSB and changes to children’s ACS, H1b) changes to children’s ACS would mediate the relationship between the WSB and changes to children’s MVPA, and H2) the WSB program would increase MVPA and decrease body mass index (BMI) z-scores over a schoolyear.

## Methods

### Study design and setting

We conducted a cluster RCT to examine the impact of a WSB intervention on child ACS. The study had a two-arm, unblinded, cluster design, comparing the intervention (WSB and transportation education materials) to education materials only as the control group, with randomization at the school level. The transportation education materials were information provided by the school district on school transportation that all students and families received. The RCT was conducted October 2012-May 2016 with two measurement points for each year. Time 1 (baseline) assessment occurred prior to randomization and Time 2 (follow-up) occurred in May or June. Year 1 took place in Houston, Texas. Years 2–4 took place in the Seattle-metro area (Washington State). This RCT was approved by the Baylor College of Medicine Institutional Review Board (IRB), Department of Research and Accountability of the Houston Independent School District, Seattle Children’s Hospital IRB, and Research, Evaluation, and Assessment Office of Seattle Public Schools. It is registered at clinicaltrials.gov (NCT01626807). The TIDieR (Template for Intervention Description and Replication) Checklist was used to describe the intervention (Supplemental File 1) [31]. 

### Participants and procedure

Study staff recruited 22 Title I-designated elementary schools in the Houston, TX and Seattle, WA metro areas through district-wide informational letters describing the study and requirements. Title I-designated schools consist of a lower-income student population and are provided with financial assistance to support educational achievement [32]. School inclusion criteria were: [1] > 60% of students qualified for the federal free/reduced lunch program [proxy for socioeconomic status (SES)] [2], non-Latino White students comprised < 50% of the student body, and [3] no existing WSB program. Study staff also identified eligible schools based on SES and race/ethnicity and targeted schools directly for recruitment. In Houston, 4 out of 4 (100%) schools that were approached participated. In Seattle, 18 out of 22 schools (82%) participated. Children were recruited by announcements, flyers, and direct recruitment by study staff through class/parent presentations and assemblies. Children were eligible if they [1] were enrolled in 3rd -5th grade (typically aged 8–11 years) at one of the study schools [2], lived within 1 mile of school or parents agreed to regularly drop off children within 1 mile of school, and [3] were physically capable of walking to/from school. Children were ineligible if another child in the household was already enrolled. Eligible children from the same household could walk with the WSB groups. Parents’ informed consent and children’s assent were obtained by distributing consent forms in English and Spanish to all 3-5th grade students. We offered a modest incentive ($2) for returning signed forms regardless of whether parents consented to the study. Parents received $20 for their and their child completing assessments and measurements at each time point ($40 total/family). Children and parents received $5 for the child completing activity measurements and returning ≥ 4 days of valid PA data at each time point ($10 total/family).

Schools were matched based on school-level SES, race/ethnicity, and total school enrollment. Schools were randomly assigned within matched pairs to intervention (*n* = 11) or control (*n* = 11) conditions. We employed a cluster randomized design, which was further stratified by study sites. Participating schools from each study site (Houston and Seattle) were randomly allocated to intervention and control arm with 1:1 allocation ratio using a random number-based algorithm.

### Intervention

The WSB program was led by trained research assistants, who would pick-up and drop-off students at designated “bus stops” near their homes and then chaperone students to/from school. Research assistants received a 4-hour field-based and classroom training, which was led by study investigators and focused on WSB and pedestrian safety. Research assistants were expected to demonstrate, teach, and undergo assessment on teaching key pedestrian safety behaviors during ACS (e.g., crossing at designated crosswalks) [33]. Children had opportunities to join the WSB at pre-determined locations along the routes (1–3 routes/school). The program was provided Monday-Friday when school was in session and lasted one school year (October/November – May/June). In general, the WSB routes were staffed on the intended school days. Children and parents decided which days were feasible to participate. The WSB program was based on the publicly available WSB Guide from the National Safe Routes to School Program in the United States [34]. 

The Ecological Model of Four Domains of Active Living guided the WSB intervention implementation and evaluation [35]. Specifically, the WSB was designed to overcome environmental barriers to ACS by providing a reliable, highly visible intervention that operates throughout the school year. WSB routes operated rain or shine, and generally avoided hazardous intersections and sections of neighborhoods with poor lighting or sidewalk maintenance. The ecological model also informed consideration of neighborhood safety (perceived environment level) and walkability (community access and characteristics level).

The Social Cognitive Theory (SCT) was used as a framework to describe likely individual-level mediators of changes to children’s ACS. It is posited that self-efficacy and outcome expectations, which are constructs of SCT, influence behavior change [36]. Therefore, examining the relationships between PA, outcome expectations, and self-efficacy can help elucidate the mechanisms through which ACS interventions impact behavior change. For SCT, the WSB intervention has several paths to increase self-efficacy and outcome expectations. For parent self-efficacy and outcome expectations, the WSB staff provides parents with confidence that their children will be able to walk to/from school, given that they are chaperoned by study staff. Further, because the WSB operates on a scheduled route like a regular school bus, parental outcome expectations are that their children will arrive safely and on time for school. For child self-efficacy, the WSB staff model and teach children how to walk safely to school. Students also took turns in the front of the ‘bus’ to practice safely crossing the streets, which in turn builds on children’s self-efficacy.

### Outcome variables

All variables were measured at the schools and pertain to individuals. The primary outcome was the percentage of days that students ACS, measured daily over five school days at both baseline and follow-up using a self-administered survey with high test-retest reliability (κ = 0.97) and convergent validity (κ = 0.87) compared to parent-report [37]. We used percentage of days (versus number of days) because some students had a shortened school week due to illness or other reasons, and this allowed for variation in the number of eligible days for commuting to school. The survey asks students, “How did you get to school today?”, with response options of school, school bus, carpool, car, metro bus, walked with an adult, walked without an adult, and biked. ACS was defined as walking or biking to school. The paper surveys were available in English and Spanish, distributed by study staff in the morning with assistance from school personnel, and completed by the students.

The secondary outcome was children’s minutes/day of MVPA, measured via accelerometry. Additional secondary exploratory outcomes were light-intensity PA and sedentary time measured via accelerometry. PA was assessed at baseline and follow-up through the ActiGraph GT3X accelerometer (ActiGraph LLC, Pensacola, FL). Research staff outfitted and trained children at school to wear the accelerometers. At each time point, students were asked to wear the accelerometer on their hip for 7 days during waking hours. Commonly used data quality standards were used to define valid wear time [38]. At least four valid days (≥ 8 h of valid wear time, including weekdays and weekends) was the goal, but not required, for inclusion in analyses [39]. Non-wear time was defined as 60 consecutive minutes or more of no data recording. Validated accelerometer cut points were used to define MVPA, light-intensity PA, and sedentary time (≥ 2296, > 100–2295, and ≤ 100 counts/min) [40]. When calculating minutes spent in different activity levels, 15-second epochs were used. Participants had a second week to wear the accelerometer should the first attempt yield inadequate valid data.

### Covariates

Assessment and intervention staff were separate to reduce potential bias. Parents and children conducted surveys on electronic devices (e.g., iPads) at schools (or by phone, if necessary), in which data were uploaded directly to a secure, encrypted server. At baseline, parents reported demographic information for themselves and their child, including age, gender, race/ethnicity, relationship to child, self-reported height and weight, educational level, household income and size, number of automobiles, and home address, which was used to estimate each participant’s distance from home to school in miles by walking by entering the address into Google maps. An 8-item parent questionnaire assessed neighborhood disorder (safety, drug traffic, violence, child victimization) on a 4-item scale (“never”, “rarely”, “sometimes”, “frequently”) at baseline (range: 0–32), with higher scores indicating higher disorder [41]. For this questionnaire, parents were asked how often they saw certain activities happening in their neighborhood, such as gang activity and disorderly groups of youths or adults.

Research staff followed a standardized protocol to measure participants’ height and weight using a portable stadiometer (Seca 214; Seca, Hamburg, Germany) and a Tanita BWB-800 S digital scale (Tanita Corporation of America, Inc, Arlington Heights, IL), respectively. Two height and weight measurements were taken at Time 1 and Time 2. If the two measures differed by more than 0.20 cm (height) or 0.20 kg (weight), a third measurement was taken. The body mass index (BMI) z-scores were calculated using participants’ average height and weight.

Additional outcomes of interest included parent and child self-efficacy and parent outcome expectations measured via questionnaires at Times 1 and 2 [20, 22, 23]. Children completed a 16-item questionnaire (Cronbach’s alpha = 0.75) to examine their self-efficacy for walking to school (e.g., “I am sure that I can walk to and from school even if it is hot outside”). Parents completed a 29-item questionnaire to examine their self-efficacy (e.g., “I am sure that I can allow my child to walk to and from school even if it is hot outside”) (15-items, Cronbach’s alpha = 0.88) and outcome expectations (e.g., “my child will be unsafe because of traffic”) (14-items, Cronbach’s alpha = 0.78) for allowing their child to walk to school [22, 23]. Three-point Likert-type scales were used to examine outcome expectations (1=“do not agree”, 2=“agree a little”, 3=“agree a lot”) and self-efficacy (1=“not sure”, 2=“a little sure”, 3=“very sure”).

### Sample size

Sample size calculations were based on detecting a change in children’s ACS in the intervention versus control group. Given a repeated measures design with two study groups, an alpha of 0.05, a correlation over time of 0.5, and a sample size of 296, there was 80% power to detect a small (Cohen’s d = 0.16) significant group by time interaction increase in ACS for the intervention group and a decrease or maintenance effect in the control group [43]. A variance inflation factor was used to account for clustering of children within schools. Assuming an ICC of 0.04 [23] and 35 children per school, the sample size needed to account for clustering was 700.

### Statistical analyses

We conducted an intention-to-treat analysis. Data were analyzed using RStudio version 4.3.1 (R Foundation for Statistical Computing, Vienna, Austria). Data were combined for both cities (Houston and Seattle), as intended. We were not powered to, nor did we intend to, examine differences by city. For our primary analysis, we used linear mixed-effect models to estimate the association between the intervention with percentage of days ACS. If a student reported that they completed the transit survey on a non-school day, that day was excluded. We adjusted for several covariates (fixed effects) including age, sex, race/ethnicity, home-to-school distance, and neighborhood disorder. We also included an exposure X time interaction term as a fixed effect. To account for within child and within school correlations, we also included random effects corresponding to correlations within students nested within schools. We used linear mixed-effects models to estimate the association between the intervention and mean (1) MVPA, (2) percentage of days ACS, (3) child self-efficacy, (4) parent self-efficacy, (5) parent outcome expectations, and (6) BMI z-scores. For the MVPA models, we also included accelerometer wear time and day of the week (weekday vs. weekend) as covariates. All days that a participant had recorded accelerometer data were included in this model.

To evaluate whether the effect of the intervention on the change in the percentage of days children ACS was mediated by change in child self-efficacy, parent self-efficacy, or parent outcome expectations, we conducted causal mediation analysis using regression-based approach by Valeri et al. (2013) and VanderWeele et al. (2014) [44, 45]. We performed these analyses using linear mixed effects regression models, as described above. Our outcome was change in percentage of days children ACS, and our mediator(s) were change in child self-efficacy, change in parent self-efficacy, and change in parent outcome expectations – examined both individually and combined. We adjusted for child age, sex, race, home-to-school distance, neighborhood disorder, and baseline percentage of days ACS. We used the {Mediation} package in R to decompose the total effect of the intervention on the change in percentage of days ACS into the average direct effect (ADE) and the average causal mediation effect (ACME, i.e., indirect effect, mediated effect) by changes in self-efficacy and outcome expectations [46]. 

We conducted a similar mediation analysis to evaluate the effect of the intervention in change in average daily MVPA mediated by the change in the percentage of days of ACS. We adjusted these models for child age, sex, race, distance from home to school, neighborhood disorder, and baseline minutes of MVPA.

## Results

### Descriptive

**Fig. 1:**
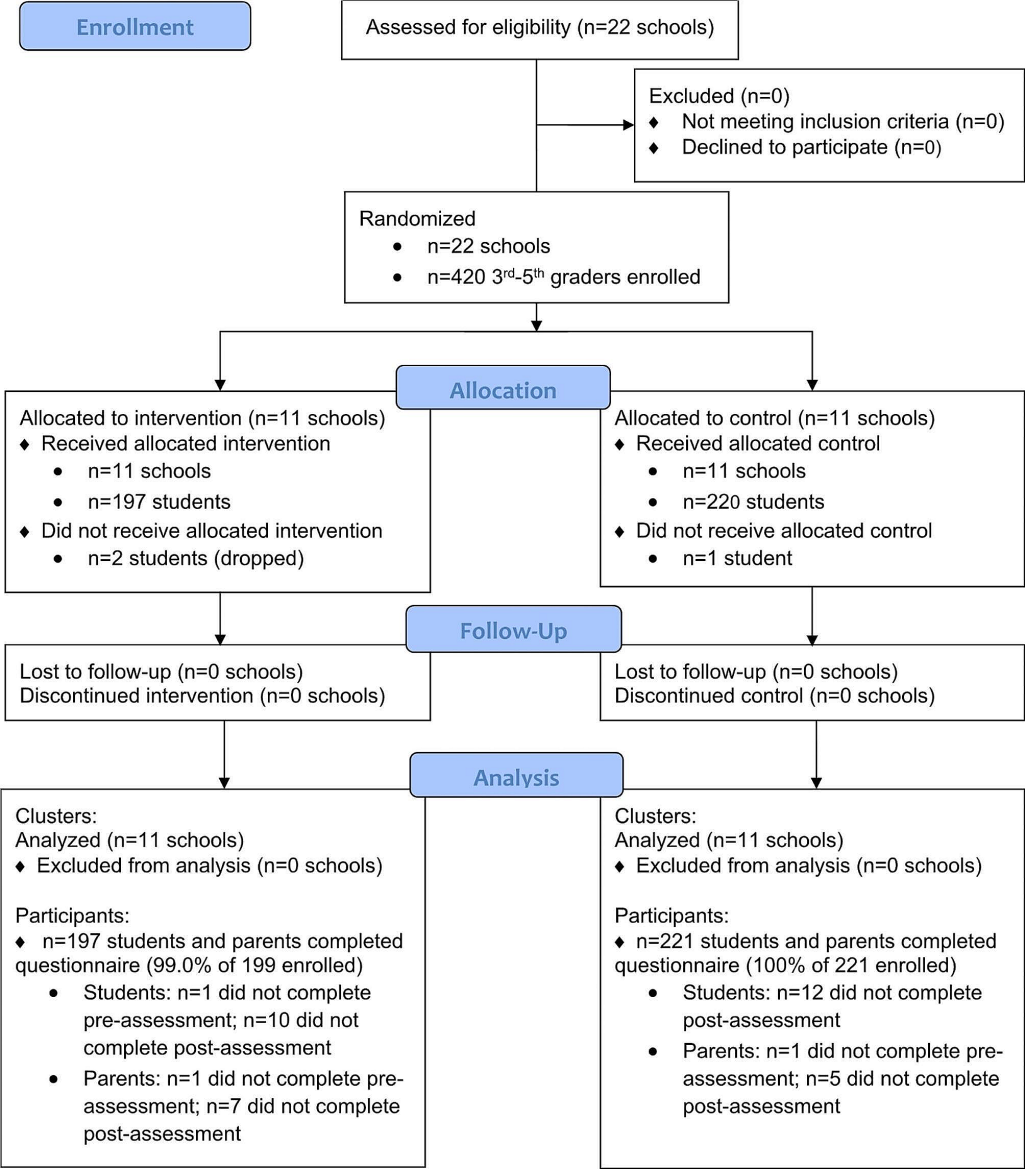
Consort diagram

Twenty-two schools participated in the cluster RCT from 2012 to 2016, each for the duration of one school year, as shown in the CONSORT diagram (Fig. 1; Supplemental File 2) [47]. A total of 418 participants and their parents enrolled in the study over the four years, with 197 in the intervention group. For child participants, the mean age was 9.2 (SD = 0.9) years, 46% were female, 28% were Latino, and the mean child BMI z score was 0.8 (SD = 1.1) (Table 1). About 23% of parents had at least a college degree and over 53% of parents reported a household income of ≤$40,000, which is below the United States median household income of $57,617 in 2016 [48]. The mean neighborhood disorder score was 14.9 (SD = 6.3; range 0–32), and the mean home-school distance was 1.0 (SD = 1.2) miles. ICCs were computed for each outcome at the school level including MVPA (0.009), ACS (0.132), child self-efficacy (0.059), parent self-efficacy (0.078), parent outcome expectations (0.025), and BMI z-score (0.063). No adverse events occurred during the study.

**Table 1:** Participant characteristics stratified by study group assignment

Characteristics	Intervention *n* = 197	Control *n* = 221	Total *N* = 417
Child age, mean (SD) (years)	9.3 ± 0.9	9.2 ± 1.0	9.2 ± 0.9
Female, n (%)	99 (50.2)	92 (41.8)	191 (45.8)
Race/Ethnicity, n (%)			
Non-Latino white	20 (10.2)	49 (22.3)	69 (16.5)
Non-Latino black	25 (12.7)	41(18.6)	66 (15.8)
Latino	58 (29.4)	58 (26.4)	116 (27.8)
Asian	32 (16.2)	21 (9.5)	53 (12.7)
Multi-racial/Other	24 (12.2)	15 (6.8)	39 (9.4)
Missing	38 (19.3)	37 (16.4)	74 (17.7)
Child BMI z-score, mean (SD)	0.8 ± 1.1	0.8 ± 1.0	0.8 ± 1.1
Distance home to school (miles), mean (SD)	0.7 ± 0.7	1.3 ± 1.5	1.0 ± 1.2
Neighborhood disorder, mean (SD)	14.0 ± 6.1	15.7 ± 6.4	14.9 ± 6.3
Parent income, n (%)			
≤$20,000	47 (23.9)	50 (22.7)	97 (23.3)
$20,001–$40,000	68 (34.5)	59 (26.8)	127 (30.5)
$40,001-$60,000	36 (18.3)	45 (20.5)	81 (19.4)
≥$60,001	45 (22.8)	65 (29.5)	110 (26.4)
Missing	1 (0.0)	2 (0.0)	2 (0.0)
Parent education, n (%)			
≤ High school	73 (37.1)	61 (27.7)	134 (32.1)
Some college or associate degree	42 (21.3)	69 (31.4)	111 (26.6)
≥ College degree	37 (18.8)	57 (25.9)	94 (22.5)
Missing	45 (22.8)	33 (15.0)	78 (18.7)

MVPA: moderate-to-vigorous intensity physical activity; BMI: body mass index; SD: standard deviation

Table 2 presents mean and SD for BMI Z-score, percentage of days ACS, MVPA, child self-efficacy, parent self-efficacy, and parent outcome expectations at baseline and post-intervention. In the unadjusted analysis, we did not detect any statistically significant between time differences between the intervention and control group from baseline to post-intervention.

**Table 2:** Unadjusted descriptive statistics for outcomes by intervention group

	Pre-intervention		Post-intervention		Between Time Differences		
	Intervention	Control	Intervention	Control	Intervention	Control	*p*-value
	Mean (SD)	Mean (SD)	Mean (SD)	Mean (SD)	Mean (SD)	Mean (SD)	
Percentage of days walking to school	36.11 (42.75)	29.25 (40.94)	44.91 (45.75)	28.64 (40.71)	7.96 (41.40)	-0.27 (28.67)	0.08
MVPA	37.10 (16.11)	38.40 (18.87)	40.87 (21.96)	39.86 (18.61)	3.62 (18.12)	1.95 (16.19)	0.77
Child self-efficacy	2.31 (0.44)	2.40 (0.45)	2.35 (0.43)	2.34 (0.44)	0.03 (0.45)	-0.08 (0.45)	0.07
Parent self-efficacy	2.25 (0.45)	2.15 (0.51)	2.23 (0.45)	2.01 (0.56)	-0.02 (0.49)	-0.11 (0.46)	0.25
Parent outcome expectations	2.09 (0.27)	2.09 (0.26)	2.10 (0.26)	2.10 (0.26)	0.01 (0.31)	-0.002 (0.27)	0.43
BMI-Z	0.83 (1.16)	0.79 (1.06)	0.75 (1.17)	0.70 (1.06)	-0.08 (0.26)	-0.07 (0.25)	0.60

SD: standard deviation; MVPA: moderate-to-vigorous intensity physical activity; BMI: body mass index; Bold indicates statistical significance at *p* < 0.05

*P*-values were based on two-sample Wilcoxon rank-sum test

### BMI z-score

Intervention children decreased their BMI z-score from time 1 to time 2 [-0.08 (95% CI: -0.11, -0.04; *p* < 0.001)]. There were similar significant decreases in BMI z-score from time 1 to time 2 among control children [-0.07 (95% CI: -0.10, -0.03; *p* < 0.001)]. The unadjusted effect of the intervention on change in BMI z-score over time was not significantly different from control arm: -0.01 (95% CI: -0.04, 0.06; *p* = 0.65).

### Mixed-effects models of intervention effects

The intervention group showed non-significant improvements in almost all outcomes from baseline to post intervention, while the control group decreased in most outcomes (Table 2). Table 3 shows the results from the adjusted regression analyses. The intervention group showed a statistically significant increase in percentage of days ACS compared to the control group over time (β = 9.04; 95% CI: 1.10, 16.98; *p* = 0.023). The intervention group also showed significant differences compared with the control group over time for daily minutes of MVPA (β = 4.31; 95% CI: 0.70, 7.91; *p* = 0.02), with significant increases shown separately for VPA (β = 1.52; 95% CI: 0.20, 2.84; *p* = 0.02) and MPA (β = 2.78; 95% CI: 0.19, 5.37; *p* = 0.04). The intervention group also showed significantly more improvement compared with the control group over time on child self-efficacy (β = 0.13; 95% CI: 0.03, 0.23; *p* = 0.01), but nonsignificant differences in parent self-efficacy (β = 0.09; 95% CI: -0.03, 0.21; *p* = 0.13), parent outcome expectations (β = 0.01; 95% CI:-0.06, 0.09; *p* = 0.70), and BMI z-score (β = 0.02; 95% CI: -0.06, 0.10; *p* = 0.62).

**Table 3:** Adjusted regression results on active commuting to school, moderate-to-vigorous intensity physical activity, child self-efficacy, parent outcome expectation outcomes, and body mass index Z-score

Outcome measures	Adjusted treatment effect (vs. Control) β (95% CI)	*P* value
Percentage of days ACS	9.04 (1.10, 16.98)	**0.03**
Daily MVPA minutes	4.31 (0.70, 7.91)	**0.02**
Child Self-efficacy	0.13 (0.03, 0.23)	**0.01**
Parent Self-efficacy	0.09 (-0.03, 0.21)	0.13
Parent Outcome Expectations	0.01 (-0.06, 0.09)	0.70
BMI Z-score	0.02 (-0.06, 0.10)	0.62

Adjusted treatment effect is the coefficient corresponding to the treatment-by-time interaction term in the linear mixed effects regression model, which captures the differences between treatment arms in terms of mean across time changes in outcomes.

All regression models adjusted for child age, child birth sex, child race/ethnicity, distance between home and school, neighborhood disorder index, and baseline values of the outcome measures. All models also included child and school-specific random effects to account for within-child and within-school correlations.

MVPA models adjusted for accelerometer wear time.

ACS: active commuting to school; MVPA: moderate-to-vigorous intensity physical activity; BMI: body mass index. Bold indicates statistical significance at *p* < 0.05.

### Mediation analyses

In our causal mediation analysis, we estimated the mediated effect using the average causal mediation effect (ACME) estimate. The effect of the intervention on change in percentage of days children were ACS was not significantly mediated by change in child self-efficacy (ACME=-0.12, *p* = 0.82), parent self-efficacy (ACME = 0.03, *p* = 0.88), or parent outcome expectations (ACME = 0.02, *p* = 0.97). The effect of the intervention on change in MVPA was not significantly mediated by change in percentage of days ACS (ACME = 0.68, *p* = 0.14), self-efficacy (ACME=-0.02, *p* = 0.93), parent self-efficacy (ACME=-0.003, *p* = 0.98), or parent outcome expectations (ACME = 0.05, *p* = 0.95).

## Discussion

This is the first known RCT to examine the long-term efficacy of a WSB program on children’s ACS, MVPA, and weight status. In this school-based cluster RCT, which enrolled students from schools that primarily serve lower income families, we found that children in the intervention group had a significant increase in the percentage of days ACS and in minutes/day of MVPA versus students in the control group. No significant differences in BMI z-scores were found between the intervention and control group over time. Changes in child self-efficacy, parent self-efficacy, and parent outcome expectations did not appear to mediate the intervention effect on change in ACS or MVPA.

The ACS and MVPA results align with findings from our pilot RCT, which show increased rates of ACS and minutes/day of MVPA among intervention students versus the control group [23]. In our pilot RCT, children in the WSB group had a relative increase of about 38% for percentage ACS and a relative increase of about 7 min/day more of MVPA versus the control [23]. Findings from quasi-experimental trials have also reported positive associations between participation in a WSB program with ACS and MVPA. For example, one study reported that > 43% of participants completely or partially changed their modes of ACS from vehicle to walking from baseline to follow-up [49], while another study demonstrated an increase in PA from a mean of 4.3 days/week to 5.3 days/week [50], although these findings were not significant. A quasi-experimental trial by Heelen et al., 2009 also demonstrated a greater percentage of children ACS among the intervention versus control schools as well as a relative increase of about 11 more minutes/day of PA among the WSB participants compared to control subjects [28]. Previous studies have demonstrated the feasibility of WSB programs, [15, 23] and the findings from this RCT provide support for the use of WSB programs to increase ACS as well as PA among elementary-aged children. Additionally, we conducted qualitative interviews postintervention to examine parent perceptions, and exercise/physical health was the most cited facilitator of participating in the program [51].

One of the study aims was to prevent excessive BMI z-score increase relative to the control group. No significant differences in BMI z-scores were seen between intervention and control children over time, although BMI z-scores decreased in both groups. Few studies have examined the effect of WSB programs on BMI, with mixed results. One quasi-experimental trial reported that BMI percentile remained stable among participants throughout the WSB program [50], and another study reported that frequent walkers across all schools gained 58% less body fat compared with passive commuters [28]. In the present study, the amount of MVPA that changed over time between groups was not large enough to produce a significant difference in BMI z-score. BMI z-score is a difficult outcome to change in children, particularly among children of normal weight, even with intensive behavioral programs. In line with the Cochrane Reviews on diet, PA, and behavioral interventions for treating overweight and obese children, multi-component interventions that incorporate diet, PA, and behavior change may be beneficial in achieving small, short‐term reductions in BMI z-score in children [52]. Still, increasing children’s PA levels through ACS may help to establish healthy behaviors early on [25] and support positive habit formation [53].

In our data, the effect of the intervention on ACS and MVPA was not mediated by change in child self-efficacy, parent self-efficacy, or parent outcome expectations, individually or combined. There are several possibilities for these findings. For the mediation of self-efficacy and outcome expectations on ACS, it is possible that the measurement of these mediators is insufficient, we lacked power for the mediation analysis, the mediators are not actual mediators, or there are other mediators lacking in the model. For the mediation of ACS on MVPA, it is possible that ACS leads to a more universal behavior change towards active living in general (i.e., beyond ACS). These additional opportunities for active living could partially or entirely contribute to mediation. It is also possible that the measurement of ACS is insufficient, or ACS is not a mediator of MVPA. The lack of significance is unsurprising given that most of the potential mediators were not found to be significantly associated with the intervention, suggesting they might not play major roles on the causal pathway. However, in a study examining the impact of our WSB program on self-efficacy and outcome expectations, the WSB improved child and parent self-efficacy, and parent outcome expectations related to ACS [20]. Additionally, improved child self-efficacy was reported by parents in the qualitative interviews [51]. The mediation findings conflict with findings from our pilot RCT, which showed that parents’ outcome expectations were a significant influence on their children’s ACS through the WSB [23]. Further, baseline results from our pilot RCT showed that parents’ self-efficacy was a significant correlate of their children’s ACS [42]. Additional research is needed to increase our understanding of the role of self-efficacy and outcome expectations in the relation between WSB program with ACS and PA.

Study generalizability should also be noted. We designed our WSB program based on the WSB guide from the National Center for Safe Routes to School program in the US [34]. It is a publicly available guide, which allows for widespread implementation, and the WSB intervention would resemble what programs look like outside of research, increasing the generalizability of the findings. For this study, like most studies, the WSB program needed to be discontinued due to lack of research funding. However, research in turn informs funding decisions and resources that may make this program available more widely.

### Limitations

Study limitations should be noted. School commuting mode was collected via self-report, and subject to recall and social desirability biases. However, we asked children directly about their commuting behavior and using a valid, reliable measure. The sample’s baseline levels of MVPA were at or slightly above national norms [38], so it is possible there was less room to increase MVPA levels and the program may be more effective in children with lower MVPA at baseline. Moreover, MVPA was measured over weekdays and weekends to capture habitual changes in MVPA. This approach may understate intervention effect on schooldays when children may ACS, although we were not powered to do an analysis focused on school day MVPA. Finally, although it was originally planned to include an objective walkability measure as a covariate [54], walkability was only measured in Seattle due to staffing limitations.

## Conclusions

This school-based cluster RCT examined the long-term efficacy of a WSB program on ACS, MVPA, and weight status among children from lower income families. Children in the intervention group had a significant increase in the percentage of days ACS, supporting hypothesis 1, and in minutes/day of MVPA versus students in the control group, partially supporting hypothesis 2. Hypotheses 1a and 1b were not supported by the findings, which showed that the effect of the intervention on ACS and MVPA was not mediated by change in child self-efficacy, parent self-efficacy, or parent outcome expectations, individually or combined. Hypothesis 2 was not fully supported, as no significant differences in BMI z-scores were seen between intervention and control children over time. The positive findings for commuting behavior and MVPA support implementation of WSB programs to be inclusive of school-age children from lower income families to support their ACS and improve PA.

### Electronic supplementary material

Below is the link to the electronic supplementary material.

Supplementary Material 1

Supplementary Material 2

## Data Availability

The datasets used and/or analyzed during the current study are available from the corresponding author on reasonable request.

## Abbreviations

US: United States
ACS: Active commuting to school
PA: Physical activity
MVPA: Moderate-to-vigorous intensity physical activity
WSB: Walking school bus
RCT: Randomized controlled trial
BMI: Body mass index
ACME: Average causal mediation effect
VPA: Vigorous intensity physical activity
ICC: Intraclass correlation
ADE: Average direct effect
SES: Socioeconomic status
